# Network analysis of burnout pathways among in-field and out-of-field math-major teachers in rural China

**DOI:** 10.3389/fpubh.2025.1635130

**Published:** 2025-09-04

**Authors:** Ming Huo

**Affiliations:** China Institute of Rural Education Development, Northeast Normal University, Changchun, China

**Keywords:** job demands-resources, job burnout, math major, in-field teachers, out-of-field teachers, network analysis

## Abstract

**Introduction:**

Teacher burnout threatens educators’ well-being and instructional quality, especially in rural schools. However, little is known about how burnout differs between in-field and out-of-field teachers with the same academic background.

**Methods:**

This study used network analysis to examine the relationships among job demands, job resources, personal resources, and burnout symptoms in 1,879 rural teachers in China with mathematics majors, including 1,682 teaching math (in-field) and 197 teaching other subjects (out-of-field).

**Results:**

Emotional exhaustion was the most central burnout symptom in both groups, with slightly higher centrality and stronger associations with job demands among out-of-field teachers. Job satisfaction consistently acted as a protective factor, particularly in reducing depersonalization. Although the overall network structures were similar, differences in node centrality and bridging patterns were observed.

**Discussion:**

These findings highlight that even among teachers with identical training, out-of-field teaching creates extra burnout risks by increasing the effects of job demands. These findings underscore the importance of reducing non-instructional burdens and supporting professional identity and efficacy to mitigate teacher burnout in order to improve the educational quality in rural schools.

## 1 Introduction

Job burnout has often been described as an individual’s emotional response to sustained and excessive work-related stress, and is particularly prevalent in professions that involve constant social interaction and emotional engagement (1, 2). This psychological syndrome is typically conceptualized through three core dimensions: emotional exhaustion, depersonalization (also referred to as cynicism), and diminished personal accomplishment (3). Emotional exhaustion reflects a state in which individuals feel physically depleted and emotionally drained due to persistent psychological demands (3). Depersonalization involves adopting a detached and indifferent attitude toward others, perceiving them as objects rather than human beings (3). The third dimension, diminished personal accomplishment, refers to a decline in self-efficacy and professional self-worth in the workplace (3). Some researchers have emphasized emotional exhaustion and/or depersonalization as the core components of burnout, with emotional exhaustion often viewed as the starting point of burnout and depersonalization as a maladaptive coping strategy to create psychological distance from exhausting demands (4–6).

Burnout has been reported among various human service professions worldwide, including police officers, doctors, and teachers (7). Evidence has shown that teachers tended to report higher levels of emotional exhaustion and depersonalization when compared to individuals in other professional fields (8). In recent years, teacher burnout has become a global concern, with growing evidence showing that teachers in many countries report moderate to high levels of burnout (86). In China, teacher burnout has become increasingly prevalent over the past two decades. Potentially driven by higher educational expectations, intensified workloads, and more rigid educational accountability systems (9, 10). A recent study in Zhejiang Province revealed that nearly 50% of primary and secondary school teachers reported experiencing severe emotional exhaustion, highlighting the scale and urgency of this issue in the Chinese educational system (11).

Rural school teachers in China face complex and demanding working conditions that increase the risk of burnout. Financial constraints, limited infrastructure, and complex social environments exacerbate the professional pressures they experience. Despite government subsidies, salaries remain low, which contributes to job dissatisfaction (12–14). Teachers are facing a dual burden of instructional and administrative responsibilities, yet having limited access to high-quality, subject-specific professional development (15, 16). The widespread phenomenon of left-behind children due to parental labor migration increases teachers’ emotional and pastoral responsibilities, often without consistent family support (17, 18). Moreover, professional isolation is quite common, with limited opportunities for peer collaboration and psychological support (13, 14, 19). Out-of-field assignments are prevalent in rural schools, adding cognitive stress and reducing pedagogical confidence (20, 21). These realities highlight that teacher burnout in rural China is not merely an individual issue but a product of broader the need to view teacher burnout in rural China not just as an individual phenomenon, but as a product of broader structural and institutional forces.

Teacher burnout has far-reaching consequences that impact not only the well-being of teachers, but also the overall quality of education and student development. Burnout has been associated with a range of physical and psychological health problems for teachers, including chronic fatigue, emotional distress, and impaired mental well-being (22–24). Teachers experiencing high levels of burnout are more likely to report lower job satisfaction, increased absenteeism, and greater intentions to leave the profession, often resulting in early retirement or career attrition (25–28). Research shows that the effects of burnout can begin early in a teacher’s career, with some studies indicating its presence as early as the student-teaching phase (29). In addition to the personal consequences, burnout impairs instructional quality and reduces teachers’ ability to provide emotional support in the classroom (30, 31), which negatively affects students’ academic achievement and social–emotional development (32, 33). Moreover, burnout has been linked to difficulties in emotional regulation (34), increased perfectionism (35), and diminished work ability (36).

To better understand the mechanisms underlying teacher burnout, the Job Demands-Resources (JD-R) model offers a comprehensive framework for examining how various aspects of the work environment influence employees’ well-being (4, 37). The JD-R model categorizes work-related factors into two broad domains: job demands, referring to the physical, emotional, or cognitive efforts required by the job, and job resources, referring to the structural or psychological supports that help individuals cope with these demands and achieve work-related goals. When job demands are high (e.g., excessive teaching loads) and job resources (e.g., peer support) are insufficient, employees may experience energy depletion and emotional strain, increasing their vulnerability to burnout (38). In addition, the JD-R model outlines two distinct processes: the health impairment process explains how long-term exposure to high job demands erodes individuals physical and emotional resources over time, leading to fatigue, stress, and ultimately burnout; and the motivational process, in which job resources enhance job performance by promoting work engagement, satisfying basic psychological needs, and fostering intrinsic motivation (39). As such, the JD-R model provides a valuable lens to identify the root causes of burnout and guide potentials interventions in the professional setting of teachers.

In addition to job demands and job resources, personal resources are increasingly recognized as protective factors within the JD-R model (40). Personal resources refer to individuals’ beliefs or self-assessment to control and influence their environment successfully (41). For instance, self-efficacy, an individual’s belief in their ability to cope with work-related challenges, has been found to be associated with low levels of burnout (42).

One significant but understudied job demand that may contribute to teacher burnout is out-of-field teaching. This is a situation in which teachers are assigned to teach subjects, year levels or school types without the necessary qualifications, certification, or specialization (43). For example, a teacher trained in mathematics but required to teach English would be considered as an out-of-field teacher. Within the JD-R framework, this form of assignment may impose significant additional demands. Teacher must invest extra time and cognitive effort to acquire unfamiliar content knowledge, adapt different teaching methods, and manage potential classroom challenges without the pedagogical confidence typically derived from subject expertise (44). These conditions may place significant demands on teachers’ emotional and mental resources, increasing the likelihood of burnout, especially when there is a lack of enough institutional support or relevant professional development opportunities (45). Furthermore, out-of-field teaching often happen in under-resourced remote or rural areas, which may further exacerbate its effects on teacher stress and instructional quality (46, 47).

Out-of-field teaching has significant negative consequences for both students and teachers. Empirical studies have shown that students taught by out-of-field teachers are more likely to receive lower-quality instruction, which can negatively affect their academic achievement and undermine equity in educational outcomes (48). At the same time, out-of-field teachers tend to report higher levels of stress, reduced teaching efficacy, and greater difficulty with classroom management (47, 49, 50), which collectively contribute to teacher burnout (51). These risks are particularly severe in rural schools, where access to mentoring, training, and peer support is often limited due to resources constraints and geographical isolation (52).

While the Job Demands–Resources (JD-R) model provides a valuable framework for understanding how work-related variables contribute to teacher burnout, most empirical studies have relied on traditional analytical methods such as regression and structural equation modeling (53). These approaches, however, typically assume linear and unidirectional relationships among variables, which limits their capacity to capture the complex and reciprocal dynamics among burnout symptoms and job-related variables. Instead of regarding burnout as a single latent construct, network analysis offers a data-driven method that conceptualizes burnout as a dynamic system of interrelated symptoms and work-related variables (54). This approach enables us to examine non-linear and multi-directional associations among job demands, job resources, and burnout dimensions. One of its key advantages lies in identifying central nodes, which refer to the variables that are most strongly connected to others and help maintain the overall network structure (55, 56). Another important feature is the detection of bridge nodes, which serve as critical links between distinct variable communities (57). In this study, variables are grouped into two communities: the JD-R cluster (including job demands, job resources, and personal resources) and the burnout cluster (including emotional exhaustion, depersonalization, and diminished personal accomplishment). By analyzing the bridge centrality between the two clusters, we can identify the most influential pathways through which the work-related conditions connect with burnout symptoms, therefore offering insights into potential targets for intervention to mitigate teacher burnout and improve occupational well-being.

Although network analysis has increasingly been used to examine teacher burnout within the JD-R framework (9, 11), studies specifically targeting rural teachers and the role of out-of-field teaching within this context remain limited. This gap is of particular concern given that schools in rural areas often experience higher rates of teacher shortages and subject mismatches (47, 58).

To address these gaps, the present study applies network analysis to investigate the interrelationships among job demands, job resources, personal resources, and burnout symptoms in rural Chinese teachers who share a common academic background in mathematics. Specifically, we focus on two groups: math majors currently teaching mathematics only (in-field) and math majors teaching subjects outside their area of expertise (out-of-field). This design allows for a more precise examination of how out-of-field teaching influences burnout experiences. There are several reasons for focusing on math majors. First, mathematics instruction often requires strong content knowledge, logical reasoning, and specialized pedagogical skills (59), which may place greater demands on teachers compared to other subjects (60). These high cognitive demands make in-field math teachers particularly susceptible to burnout due to the intense mental and instructional effort needed to support students’ understanding. Second, math majors teaching out-of-field may lack confidence in instructional strategies for unfamiliar subjects, which may force them to spend more time on lesson preparation, reduce their instructional efficacy, and increase emotional strain. Third, by selecting teachers with a common academic background (mathematics) but differing current teaching assignments, this study controls for variations in disciplinary training. This allows for a more focused investigation of how out-of-field teaching influences burnout mechanisms: whether burnout arises from the complexity of math instruction itself or from the challenges of out-of-field teaching.

By identifying the central and bridge nodes within the burnout networks, this study aims to reveal the most influential factors contributing to teacher burnout in rural settings. Through a comparative network approach, we seek to understand how the structure and dynamics of burnout differ between in-field and out-of-field math majors. Specifically, we address the following research questions:

1. What are the most central nodes within the networks of job demands, job resources, personal resources, and burnout symptoms for in-field and out-of-field math majors, respectively?
2. Which variables serve as bridge nodes between the burnout cluster and the JD-R cluster for each teacher group?
3. Are there significant differences in overall network structure and global connectivity between in-field and out-of-field math majors?

## 2 Materials and methods

### 2.1 Participants and procedures

This study utilized data from the 2018 National Survey of Teaching Workforce in Rural Areas, a large-scale survey carried out by the China Institute of Rural Educational Development. The sampling strategy involved randomly selecting 35 counties from 18 provinces across China. Within each selected county, half of the towns were randomly chosen, and all lower secondary school teachers in those towns were invited to complete an online questionnaire via the Wenjuanxing platform^1^ during the period of April to July 2018.

Of the 26,531 teachers invited from 351 schools, a total of 20,858 teachers from 341 schools completed the survey. For the purposes of this study, we identified a subsample of 1,879 lower secondary teachers who held a university degree in mathematics. Among them, 1,682 teachers were categorized as in-field mathematics majors, defined as math graduates who were currently teaching mathematics only. The remaining 197 were categorized as out-of-field mathematics majors, defined as teachers with a mathematics degree who were currently teaching subjects other than mathematics (e.g., physics, biology, or chemistry).

### 2.2 Measures

The teacher questionnaire included both fixed-response items and Likert-scale questions assessing a broad range of constructs, including job demands, job resources, personal resources, burnout, and professional demographics. All measurement instruments were translated into Chinese and adapted for contextual relevance by bilingual educational researchers. Demographic and professional information are summarized in Table 1 and descriptive statistics for all continuous variables are presented in Table 2.

**Table 1:** Description of sociodemographic and professional characteristic of participants.

Variables	In-field (*n* = 1,682)	Out-of-field (*n* = 197)
Age (years)	37.9 (8)	40.2 (8.9)
Gender		
Male	913 (54.3%)	114 (57.9%)
Female	769 (45.7%)	83 (42.1%)
Ethnicity		
Han	1,556 (92.5%)	185 (93.9%)
Ethnic minority	126 (7.5%)	12 (6.1%)
Marital status		
Unmarried	201 (12%)	20 (10.2%)
Married	1,434 (85.3%)	169 (85.8%)
Divorce/Widowed	47 (2.8%)	8 (4.1%)
Years of Teaching	15 (8.7)	17.5 (9.3)
Initial Degree		
Graduate	7 (0.4%)	1 (0.5%)
Undergraduate	605 (36%)	32 (16.2%)
Junior college	1,070 (63.6%)	164 (83.2%)
Professional Title		
Senior	248 (9.6%)	42 (21.3%)
First-grade	636 (37.8%)	70 (35.5%)
Second-grade	668 (39.7%)	66 (33.5%)
Third-grade and below	27 (1.6%)	9 (4.5%)
No professional title	103 (6.1%)	10 (5.1%)

Standard deviation in parenthesis.

**Table 2:** Descriptive statistics of all variables (means and standard deviations) for in-field and out-of-field math-major teachers.

Variable	Short codes	In-field		Out-of-field		Cronbach α
		Mean	SD	Mean	SD	
Average teaching hours per week	JD1	13.35	4.77	11.92	4.77	NA
Stress from student management	JD2	6.27	2.01	6.39	1.86	0.92
Stress from workload	JD3	7.51	1.5	7.42	1.47	0.74
Collaboration among teachers	JR1	3.84	0.84	3.81	0.82	0.83
Teacher-student relationship	JR2	3.52	0.9	3.58	0.82	0.91
School resources	JR3	3.33	0.98	3.35	0.94	0.79
School environment	JR4	2.93	0.83	2.91	0.72	0.86
Organizational justice	JR5	3.27	0.95	3.33	0.89	0.88
Job satisfaction	JR6	3.23	0.93	3.25	0.94	0.76
Classroom management efficacy	PR1	7.01	1.5	6.84	1.55	0.95
Instructional efficacy	PR2	7.27	1.34	7.21	1.39	0.94
Emotional exhaustion	B1	4.3	1.15	4.1	1.17	0.74
Depersonalization	B2	3.13	1.34	2.94	1.28	0.84
Diminished personal accomplishment	B3	3.59	1.34	3.36	1.29	0.77

SD, standard deviation.

#### 2.2.1 Job demands

Three variables were used to assess job demands: average weekly teaching hours, stress from student management, and workload-related stress. Teachers self-reported their typical weekly teaching hours. Perceived stress from student behavior (4 items; e.g., “This class is difficult to manage”) and perceived stress from workload (2 items; e.g., “There is too much work such as lesson preparation and grading”) were adapted from the Teacher Stress Inventory (61). Responses were rated on a 9-point Likert scale ranging from 1 (Not at all stressful) to 9 (Extremely stressful).

#### 2.2.2 Job resources

Job resources were measured using six variables: teacher collaboration, teacher–student relationships, school resources, general school environment, perceived organizational justice, and job satisfaction. Items assessing teacher collaboration (3 items), teacher-student relationships (3 items), and school resources (2 items) were derived from the Revised School-Level Environment Questionnaire (62). School environment (4 items), organizational justice (3 items), and job satisfaction (3 items) were based on the Teaching and Learning International Survey (63). All items were rated using a 5-point Likert scale from 1 (Strongly disagree) to 5 (Strongly agree).

#### 2.2.3 Personal resources

Two aspects of teacher self-efficacy were used to represent personal resources: instructional efficacy and classroom management efficacy. Each construct was measured using four items adapted from the Teachers’ Sense of Efficacy Scale (64). Sample items included “To what extent can you craft effective questions for your students?” (instructional efficacy) and “How much can you do to get students to follow classroom rules?” (classroom management efficacy). Items were rated on a 9-point scale ranging from 1 (Not at all capable) to 9 (Highly capable).

#### 2.2.4 Burnout

Burnout symptoms were assessed using the nine-item Bergen Burnout Inventory (BBI-9) (65), which was selected because it is brief and conceptually aligned with the three-dimensional model of burnout. The BBI-9 includes three items for each of the following dimensions: emotional exhaustion (e.g., “I often sleep poorly because of the circumstances at work.”), depersonalization (e.g., “I feel dispirited at work and I think of leaving my job”), and diminished personal accomplishment (e.g., “I frequently question the value of my work”). Each item was rated on a 6-point Likert-type scale from 1 (To a very low degree) to 6 (To a very high degree). Subscale scores were computed by averaging item responses, with higher scores indicating greater severity of burnout.

### 2.3 Data analysis

To examine the interrelationships among job demands, job resources, personal resources, and burnout, we adopted a network analytic approach using R (version 4.3.2 in RStudio 2023.12.0 + 369). The analytic process included four main steps: (1) network estimation and visualization, (2) centrality and bridge centrality computation, (3) network accuracy and stability evaluation, and (4) group-level network comparison.

#### 2.3.1 Network estimation

We estimated the network structure based on the Gaussian Graphical Model (GGM) (66). To control for the effects of demographic and professional characteristics, including age, sex, ethnicity, educational background, marital status, teaching experience, and professional title, each of the 14 study variables was regressed on these covariates. The residuals from these regressions were then used as input for network estimation. This approach allowed us to isolate the associations among core study variables while minimizing potential confounding effects from teachers’ background and professional development characteristics.

To address non-normality, a nonparanormal transformation was applied via the *huge* package (67). The GGM was estimated with the *bootnet* package (66) using the graphical least absolute shrinkage and selection operator (GLASSO) (68) and model selection via the extended Bayesian Information Criterion (EBIC) (69). Networks were visualized using the *qgraph* package (70), where thicker edges represent stronger partial correlations. Edge color represent the direction of the association (blue = positive; red = negative).

#### 2.3.2 Node and bridge centrality

To identify the most influential variables in the networks (Research Question 1), we computed expected influence (EI), the sum of all edge weights connected to a given node, taking into account both positive and negative associations (71). This measure reflects the overall impact of each variable within the system. To examine how the variables in JD-R cluster are connected to the burnout dimensions (Research Question 2), we calculated bridge expected influence (bridge EI), which quantifies the extent to which a node in one community (e.g., the JD-R cluster) connects to nodes in another (e.g., the burnout cluster). Node centrality and bridge centrality indices were computed using the *qgraph* package (66) and *networktools* package (72) respectively.

#### 2.3.3 Network accuracy and stability

To ensure the reliability of the network analysis, we evaluated the accuracy of edge weights and the stability of centrality measures using the *bootnet* package (66). First, edge weight accuracy was examined via non-parametric bootstrap resampling with 3,000 iterations to generate 95% confidence intervals (CIs), where narrower CIs indicate greater estimation precision. Second, in order to assess the stability of EI and bridge EI indices, we employed was a case-dropping bootstrap procedure. This method involves repeatedly removing incremental proportions of the sample and assessing whether centrality estimates remain consistent across these subsets The correlation stability coefficient (CS-coefficients) quantifies the correlation between centrality indices from the full sample and those from resampled subsets. A CS-coefficient above 0.25 is considered acceptable, while values exceeding 0.5 indicate strong stability (66).

#### 2.3.4 Network comparison

To address Research Question 3, network comparison tests (NCTs) (73) were conducted using the *NetworkComparisonTest* package to test for differences in the network structure (i.e., the pattern of node connections) and global strength of connections (i.e., sum of absolute edge weights) between the two teacher groups. This comparison helped identify whether and how the structure of burnout-related mechanisms differed between teacher groups.

## 3 Results

### 3.1 Network structure and visualization

**Figure 1:**
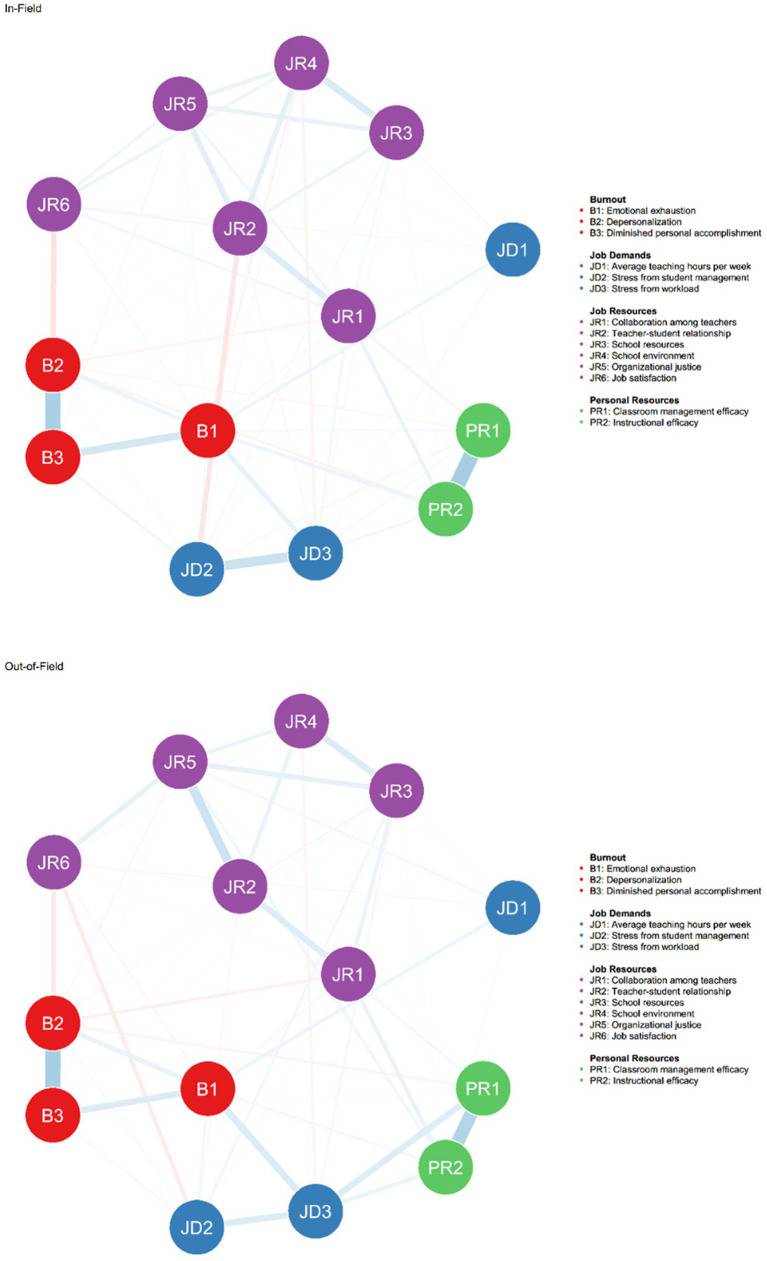
Estimated networks of job demands, job resources, personal resources, and burnout for in-field and out-of-field math-major teachers respectively. Blue edges indicate positive weights; red edges indicate negative weights. All variables in these estimated networks have been adjusted for the following covariates: age, gender, ethnicity, marital status, yeas of teaching, educational background and professional title.

The estimated networks for in-field and out-of-field math major teachers are presented in Figure 1. Of the 91 possible edges among the 14 variables, the in-field network included 68 non-zero edges (74.7%), whereas the out-of-field network contained 62 non-zero edges (68.1%). The complete edge weights for both networks are reported in Supplementary Tables 1 and 2.

For the in-field network, edge weights ranged from −0.20 (between depersonalization (B2) and job satisfaction (JR6)) to 0.66 (between classroom management efficacy (PR1) and instructional efficacy (PR2)). For the out-of-field network, edge weights ranged from −0.14 (between depersonalization (B2) and job satisfaction (JR6)) to 0.59 (between depersonalization (B2) and diminished personal accomplishment (B3)).

Within the burnout domain, strong associations were observed among the three burnout symptoms. Emotional exhaustion (B1) was positively linked with depersonalization (B2) and diminished personal accomplishment (B3) in both groups, with edge weights of 0.14 and 0.31 (in-field), and 0.15 and 0.23 (out-of-field), respectively. Depersonalization (B2) was also strongly connected to diminished personal accomplishment (B3), particularly in the in-field network (0.64) and slightly less so in the out-of-field network (0.59).

Among job demands, workload stress (JD3) was consistently associated with emotional exhaustion (B1), with edge weights of 0.18 (in-field) and 0.23 (out-of-field), indicating that high workload is a common contributor to teacher burnout across both groups. Additionally, stress from student management (JD2) was moderately associated with burnout symptoms, especially with diminished personal accomplishment (B3) in both groups (0.10 for in-field; 0.06 for out-of-field).

Regarding job resources, job satisfaction (JR6) exhibited a protective effect on depersonalization, showing negative associations of −0.20 (in-field) and −0.14 (out-of-field). Several other job resources (e.g., organizational justice, teacher-student relationships) also showed varying degrees of associations with burnout dimensions. In both networks, instructional efficacy (PR2) and classroom management efficacy (PR1) were strongly connected (0.66 in-field, 0.51 out-of-field), suggesting consistent internal alignment between personal resources.

Overall, the network structure revealed similar main associations across groups but also suggested some differences in how various JD-R variables interact with burnout dimensions in in-field versus out-of-field teachers. These differences were further examined in subsequent network centrality and comparison analyses.

### 3.2 Node centrality

**Figure 2:**
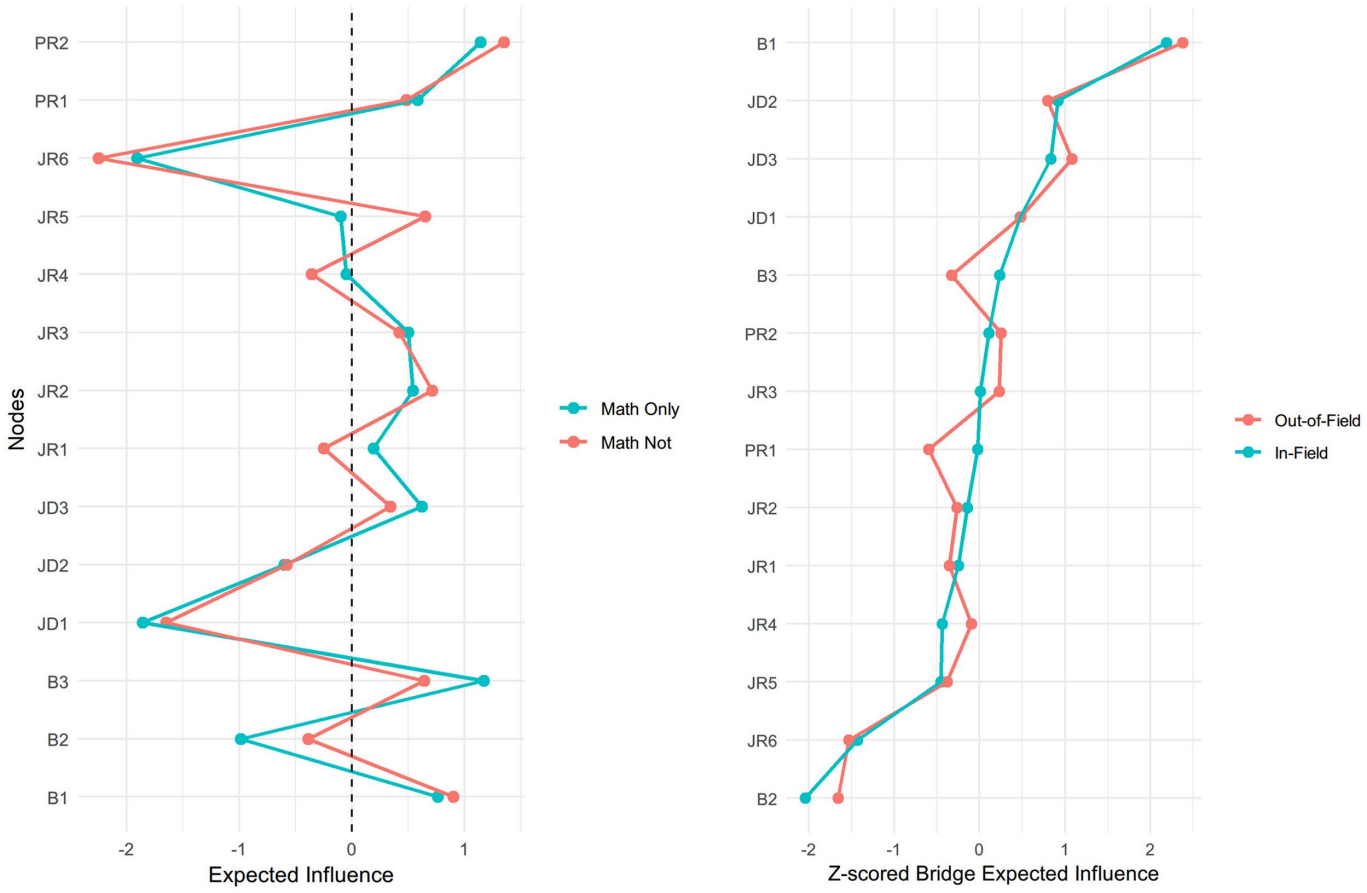
Standardized EI values among in-field and out-of-field math-major teachers. Bridge EI values of all variables for in-field and out-of-field math-major teachers.

Tables 3, 4, along with Figure 2, present raw and standardized expected influence (EI) values for all nodes in the networks of in-field and out-of-field math-major teachers, enabling comparison of node centrality within each network. In the in-field network, diminished personal accomplishment (B3) exhibited the highest EI in both raw (1.01) and standardized (1.17) forms, followed by instructional efficacy (PR2; raw: 1.01, standardized: 1.14). These variables were central largely due to their strong positive associations with other personal and burnout-related factors. Emotional exhaustion (B1) also ranked highly (raw: 0.90, standardized: 0.76), reflecting its strong connections with both job demands and other burnout dimensions.

**Table 3:** Raw expected influence (EI) values of all variables for in-field and out-of-field math-major teachers.

Variable	Short codes	Expected influence	
		In-field	Out-of-field
Emotional exhaustion	B1	**0.90 (3)**	**0.86 (2)**
Depersonalization	B2	0.40 (12)	0.43 (11)
Diminished personal accomplishment	B3	**1.01 (1)**	**0.77 (5)**
Average teaching hours per week	JD1	0.15 (13)	0.02 (13)
Stress from student management	JD2	0.51 (11)	0.37 (12)
Stress from workload	JD3	**0.86 (4)**	0.67 (8)
Collaboration among teachers	JR1	0.73 (8)	0.48 (9)
Teacher-student relationship	JR2	0.83 (6)	**0.80 (3)**
School resources	JR3	0.82 (7)	0.70 (7)
School environment	JR4	0.66 (9)	0.44 (10)
Organizational justice	JR5	0.65 (10)	**0.78 (4)**
Job satisfaction	JR6	0.13 (14)	−0.18 (14)
Classroom management efficacy	PR1	**0.84 (5)**	0.72 (6)
Instructional efficacy	PR2	**1.01 (2)**	**1.01 (1)**

Rank order in parentheses; top five in bold.

**Table 4:** Standardized expected influence (EI) values of all variables for in-field and out-of-field math-major teachers.

Variable	Short codes	Expected influence	
		In-field	Out-of-field
Emotional exhaustion	B1	**0.76 (3)**	**0.9 (2)**
Depersonalization	B2	−0.99 (12)	−0.39 (11)
Diminished personal accomplishment	B3	**1.17 (1)**	**0.64 (5)**
Average teaching hours per week	JD1	−1.86 (13)	−1.65 (13)
Stress from student management	JD2	−0.6 (11)	−0.58 (12)
Stress from workload	JD3	**0.62 (4)**	0.34 (8)
Collaboration among teachers	JR1	0.19 (8)	−0.25 (9)
Teacher-student relationship	JR2	0.54 (6)	**0.71 (3)**
School resources	JR3	0.5 (7)	0.42 (7)
School environment	JR4	−0.05 (9)	−0.36 (10)
Organizational justice	JR5	−0.1 (10)	**0.65 (4)**
Job satisfaction	JR6	−1.91 (14)	−2.25 (14)
Classroom management efficacy	PR1	**0.58 (5)**	0.48 (6)
Instructional efficacy	PR2	**1.14 (2)**	**1.35 (1)**

Rank order in parentheses; top five in bold.

Although depersonalization (B2) had a negative standardized EI (−0.99), indicating lower relative centrality, its raw EI was positive (0.40), suggesting overall positive associations with other nodes. The negative standardized score reflects its lower relative significance compared to other variables, but not the presence of negative associations.

In the out-of-field network, instructional efficacy (PR2) was the most central node, with the highest EI in both raw (1.01) and standardized (1.35) scores, primarily due to its strong positive association with classroom management efficacy (PR1). Emotional exhaustion (B1) followed closely (raw: 0.86, standardized: 0.90), highlighting its central role in the out-of-field teaching context. Other high-ranking variables included teacher-student relationship (JR2; raw: 0.80, standardized, 0.71), organizational justice (JR5; raw: 0.78, standardized, 0.65), and classroom management efficacy (PR1; raw: 0.72, standardized: 0.48), highlighting the critical role of job and personal resources in this network.

Similar to the in-field network, depersonalization (B2) exhibited a negative standardized EI (−0.39) but a positive raw EI remained (0.43), indicating lower relative centrality while maintaining positive associations with other nodes. Job satisfaction (JR6) had the lowest EI in the out-of-field network (raw: −0.18, standardized: −2.25), highlighting its role as a key protective factor despite limited direct associations with other variables.

### 3.3 Bridge centrality

As noted earlier, the network was divided into two communities: a burnout cluster (including emotional exhaustion (B1), depersonalization (B2), and diminished personal accomplishment (B3)) and a JD-R cluster comprising job demands, job resources, and personal resources. To identify potential intervention targets that connect these communities, we computed both raw and standardized bridge expected influence (bridge EI). Raw bridge EI (Table 5) quantifies the actual strength and direction of cross-cluster connections. Standardized bridge EI (Table 6), expressed as z-scores, identifies nodes that are relatively more central as bridges within each network.

**Table 5:** Raw bridge expected influence (EI) values of all variables for in-field and out-of-field math-major teachers.

Variable	Short codes	Bridge expected influence	
		In-field	Out-of-field
Emotional exhaustion	B1	**0.45 (1)**	**0.48 (1)**
Depersonalization	B2	−0.38 (14)	−0.30 (14)
Diminished personal accomplishment	B3	0.07 (5)	−0.05 (9)
Average teaching hours per week	JD1	**0.11 (4)**	**0.11 (4)**
Stress from student management	JD2	**0.20 (2)**	**0.17 (3)**
Stress from workload	JD3	**0.18 (3)**	**0.23 (2)**
Collaboration among teachers	JR1	−0.03 (10)	−0.05 (9)
Teacher-student relationship	JR2	−0.01 (9)	−0.03 (8)
School resources	JR3	**0.02 (7)**	0.06 (6)
School environment	JR4	−0.07 (11)	0.0 (7)
Organizational justice	JR5	−0.07 (11)	−0.06 (11)
Job satisfaction	JR6	−0.26 (13)	−0.28 (13)
Classroom management efficacy	PR1	0.01 (8)	−0.10 (12)
Instructional efficacy	PR2	0.04 (6)	**0.07 (5)**

Rank order in parentheses; top five in bold.

**Table 6:** Standardized bridge expected influence (EI) values of all variables for in-field and out-of-field math-major teachers.

Variable	Short codes	Bridge expected influence	
		In-field	Out-of-field
Emotional exhaustion	B1	**2.16 (1)**	**2.32 (1)**
Depersonalization	B2	−2.01 (14)	−1.62 (14)
Diminished personal accomplishment	B3	0.24 (5)	−0.32 (9)
Average teaching hours per week	JD1	**0.47 (4)**	**0.47 (4)**
Stress from student management	JD2	**0.91 (2)**	**0.78 (3)**
Stress from workload	JD3	**0.82 (3)**	**1.05 (2)**
Collaboration among teachers	JR1	−0.25 (10)	−0.35 (10)
Teacher-student relationship	JR2	−0.14 (9)	−0.28 (8)
School resources	JR3	**0.01 (7)**	0.22 (6)
School environment	JR4	−0.44 (11)	−0.10 (7)
Organizational justice	JR5	−0.45 (12)	−0.37 (11)
Job satisfaction	JR6	−1.41 (13)	−1.50 (13)
Classroom management efficacy	PR1	−0.02 (8)	−0.58 (12)
Instructional efficacy	PR2	0.11 (6)	**0.24 (5)**

Rank order in parentheses; top five in bold.

In both networks, emotional exhaustion (B1) was the strongest bridge from the burnout cluster to the JD-R cluster (raw: 0.45 in-field, 0.48 out-of-field; standardized: 2.16 in-field, 2.32 out-of-field). This indicates that emotional exhaustion serves as a critical point of interaction through which JD-R conditions may impact or amplify other burnout symptoms. Conversely, depersonalization (B2) showed the lowest standardized bridge EI (−2.01 in-field; −1.62 out-of-field), indicating a minimal bridging role. Its raw bridge EI was also moderately negative (−0.38 and −0.30, respectively), reflecting limited and suppressive cross-cluster interactions rather than an important bridging function.

Within the JD-R cluster, job demands exhibited significant bridging roles. Specifically, stress from workload (JD3) and stress from classroom management (JD2) demonstrated consistently high bridge EI values in both networks (JD3: raw 0.18 in-field, 0.23 out-of-field; standardized 0.82 in-field, 1.05 out-of-field; JD2: raw 0.20 in-field, 0.17 out-of-field; standardized 0.91 in-field, 0.78 out-of-field). These findings indicate that the two types of stress are likely key transmission pathways through which working conditions may activate or intensify burnout symptoms, especially emotional exhaustion. Additionally, average teaching hours (JD1) also showed moderate bridging effects (raw: 0.11 in both networks; standardized: 0.47 in both networks), suggesting a less significant but still meaningful role in connecting job demands to burnout symptoms.

Most job resources exhibited negative standardized bridge EI, indicating their limited bridging roles across the two clusters. For example, job satisfaction (JR6) had low raw bridge EI values (−0.26 in-field, −0.28 out-of-field), and even lower standardized scores (−1.41 in-field, −1.50 out-of-field), suggesting weak connections to burnout symptoms. Interestingly, while instructional efficacy (PR2) had high overall centrality, its raw bridge EI was modest (0.04 in-field, 0.07 out-of-field), and its standardized bridge EI also ranked moderately (0.11 in-field, 0.24 out-of-field). This indicates that instructional efficacy primarily supports within-cluster interactions rather than linking JD-R factors to burnout symptoms.

### 3.4 Network accuracy and stability

Edge-weight bootstrapping results (see Supplementary Figure 1) demonstrated that both networks were estimated with reasonable precision. The 95% confidence intervals around edge weights were relatively narrow, suggesting that the estimated associations between variables are reliable. To assess the robustness of centrality estimates, we calculated the correlation stability (CS) coefficients for expected influence (see Supplementary Figure 2). For the in-field network, the CS-coefficient was 0.75, indicating high stability and strong confidence in the centrality rankings. In contrast, the out-of-field network exhibited a lower CS-coefficient of 0.284, reflecting moderate stability and suggesting greater caution in interpreting centrality results for this group. This lower stability is likely attributable to the smaller sample size of out-of-field math majors, which can limit the precision of network estimates.

### 3.5 Network comparison

The Network Comparison Test (NCT) revealed no significant differences in the overall network structure between in-field and out-of-field math majors (*M* = 0.17, *p* = 0.31). Consequently, we did not conduct further analyses on specific edge differences between the networks. Similarly, no significant differences were observed in global strength (i.e., the total connectivity in the network): in-field group = 6.88, out-of-field group = 5.90, difference = 0.97, *p* = 0.41. These results suggest that despite group differences in individual node centrality and bridge influence, the overall configuration and connectivity of the burnout networks were comparable across the two teacher groups.

## 4 Discussion

### 4.1 Centrality of the JD-R and burnout network (RQ1)

Across both in-field and out-of-field networks, emotional exhaustion (B1) emerges as a central driver of burnout, consistent with its established role as a core symptom (1, 4, 74) and particularly its connections to work-related pressures, driving other burnout symptoms in both groups. While instructional efficacy (PR2) holds a significant role in both networks, its high general centrality (raw and standardized EI) suggests it sustain internal JD-R dynamics, but its modest raw bridge EI indicates limited direct influence on burnout dimensions.

However, distinct centrality patterns reveal unique burnout mechanisms for each group. For in-field math teachers, math majors teaching within their expertise, diminished personal accomplishment (B3) emerged as the most central node, reflecting its strong associations with emotional exhaustion (B1) and depersonalization (B2). Unlike their out-of-field counterparts, its weak connections to JD-R variables indicate it grows as a self-reinforcing symptom, amplified by emotional exhaustion and depersonalization within their specialized domain. This pattern is in line with the previous finding that diminished personal accomplishment typically arises as a consequence of emotional exhaustion or depersonalization, rather than being directly triggered by work-related demands or supports (75).

In contrast, the out-of-field network, where math majors teach non-math subjects, highlights organizational justice (JR5) as a relatively central job resource. This centrality, more significant than in the in-field network, reflects moderate-to-strong connections to teacher-student relationship (JR2), school resources (JR3), and job satisfaction (JR6), suggesting an integrative role within the job resource system. For these teachers, perceived fairness may offer procedural support and a sense of inclusion, critical for coping with the identity disruption of out-of-field teachers (76). It is important to note that the CS-coefficient for the out-of-field network was 0.284, indicating only moderate stability. Therefore, interpretations of centrality results for this group should be made with caution.

### 4.2 Bridge pathways between JD-R variables and burnout (RQ2)

In the burnout cluster, bridge EI analysis revealed that emotional exhaustion (B1) is the primary pathway linking with job demand, job resources, and personal resources. In the JD-R cluster, three job demands, stress from workload (JD3), stress from classroom management (JD2), and average teaching hours per week (JD1), together with one job resource variable that is job satisfaction, also serve as critical bridges, reflecting their strong connections to burnout dimensions. These demands and resource are classic factors influencing teacher burnout (77–80), and are especially relevant in rural Chinese education.

In rural China, teachers are often required to teach multiple subjects and grade levels, particularly in deeply impoverished areas (81). Beyond classroom instruction, they also undertake numerous non-teaching responsibilities such as boarding supervision, poverty data collection, school administration, and teacher evaluations, which require significant physical and emotional labor (13, 14, 82–84). In addition, rural teachers often have to deal with the living and emotional needs of their students with limited institutional support, which may amplify workload and emotional strain and increase burnout risk (85). One unique contribution of this study is confirming stress from workload (JD3) as a stronger bridge for out-of-field teachers and stress from student management (JD2) more evident for in-field teachers.

Apart from the three job demands, job satisfaction acts as a key protective factor, particularly in mitigating depersonalization. This protective effect aligns with the JD-R model’s motivational process, suggesting that positive attitudes and perceived rewards can help rural teachers cope with physical and psychological strain. In contrast, other job resources, such as teacher-student relationship, organizational justice and school resources, showed weak or negligible bridge influence, indicating their limited protective effects in high-demand and resource-scarce setting like rural schools. This implies that in such environments, the strain from excessive workload may diminish the impact of these resources, highlighting the need to prioritize implementing burden reduction measures and enhancing job satisfaction as core strategies to mitigate burnout.

### 4.3 Comparing in-field and out-of-field burnout networks (RQ3)

Network comparison tests revealed no significant differences in overall structure or global strength between the in-field and out-of-field teachers, suggesting that the core burnout mechanisms are shaped by shared challenges in rural Chinese schools, such as heavy workloads and limited access to professional and institutional support (13, 14, 81, 85). However, node-level patterns, such as the higher bridge role of workload stress (JD3) among out-of-field teachers, may indicate possible differences in how burnout is experienced. Since the network comparison tests revealed no statistically significant differences in structure or global strength, and the bootstrapped confidence intervals for edge weights were relatively wide, these observed differences should be interpreted with caution and viewed as potential rather than definitive differences between the two groups. These patterns suggest that, despite a shared network framework, out-of-field teaching may introduce unique stressors that could require tailored support, such as subject-specific training or workload reduction, to enhance resilience and alleviate the risk of burnout.

### 4.4 Limitations

Several limitations of this study should be acknowledged. First, the use of cross-sectional and self-reported data restricts our ability to draw causal conclusions and may introduce common method bias. Future research should employ longitudinal designs and include multiple data sources to capture the evolving nature of burnout and its underlying mechanisms more accurately. Second, while the network analysis focused on job demands, job resources, and personal resources, it did not include other potentially factors such as teacher resilience or coping strategies, serving as burnout buffers. Future research should integrate these elements to provide a more comprehensive view of how protective factors operate within the JD-R framework. Third, this study was conducted in the context of rural China, where teachers often experience unique challenges such as limited career mobility, secured tenure, and limited access to professional development. These contextual features may constrain the generalizability of our findings to urban schools or international contexts. Further research is needed to validate these burnout mechanisms across diverse educational settings. Finally, although we statistically controlled for background characteristics such as teaching experience and professional title by calculating residuals, this approach does not allow us to examine how these covariates may interact with the network structure itself. Future research could explore how teacher characteristics moderate the dynamics of burnout using moderated network models or subgroup comparisons.

## 5 Conclusion

Using network analysis within the JD-R framework, this study mapped the interconnections between job demands, job resources, personal resources, and burnout symptoms among in-field and out-of-field teachers with a math major in rural China. Emotional exhaustion was identified as the central burnout symptom, with three job demands, stress from workload, stress from classroom management, and average teaching hours, acting as critical bridges from the JD-R cluster to the burnout cluster. In contrast, job satisfaction consistently acted as a buffer, particularly against depersonalization. These findings highlight the need for targeted interventions that alleviate workload pressures and enhance job satisfaction to mitigate burnout in rural schools. While out-of-field teaching does not fundamentally change the burnout network structure, it appears to intensify existing stressors—particularly those related to workload. Addressing the needs of out-of-field teachers therefore requires more than just retraining; it also calls for structural changes that reduce their exposure to high-pressure demands. Combining subject-specific training with broader workload reduction strategies—such as employing support staff for non-instructional duties or refining teacher assignment policies—could help alleviate the cumulative pressures faced by this group.

## Data Availability

The original contributions presented in the study are included in the article/Supplementary material, further inquiries can be directed to the corresponding author.

## Footnotes

^1^ www.wjx.cn
