# Supplementary material for: Network analysis of burnout pathways among in-field and out-of-field math-major teachers in rural China
Source: Front Public Health. 2025 Sep 4;13:1635130. doi: 10.3389/fpubh.2025.1635130 (PMC12443697; doi:10.3389/fpubh.2025.1635130)
Supplement: Supplementary file 1 [file Data_Sheet_1.docx]

Supplementary Material

# Supplementary Figures and Tables

## Supplementary Figures


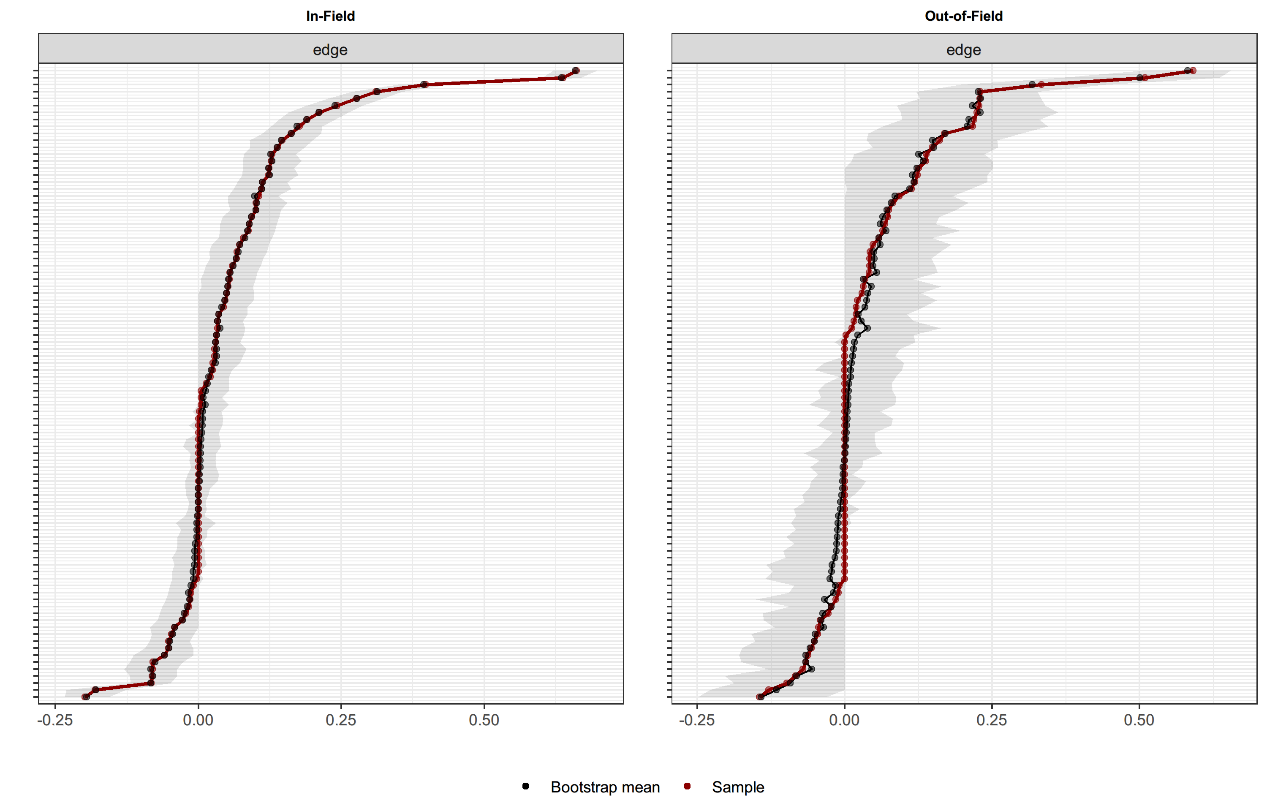


**Supplementary Figure 1.** Bootstrapped 95% confidence intervals of the edge weights for in-field and out-of-field math-major teachers.


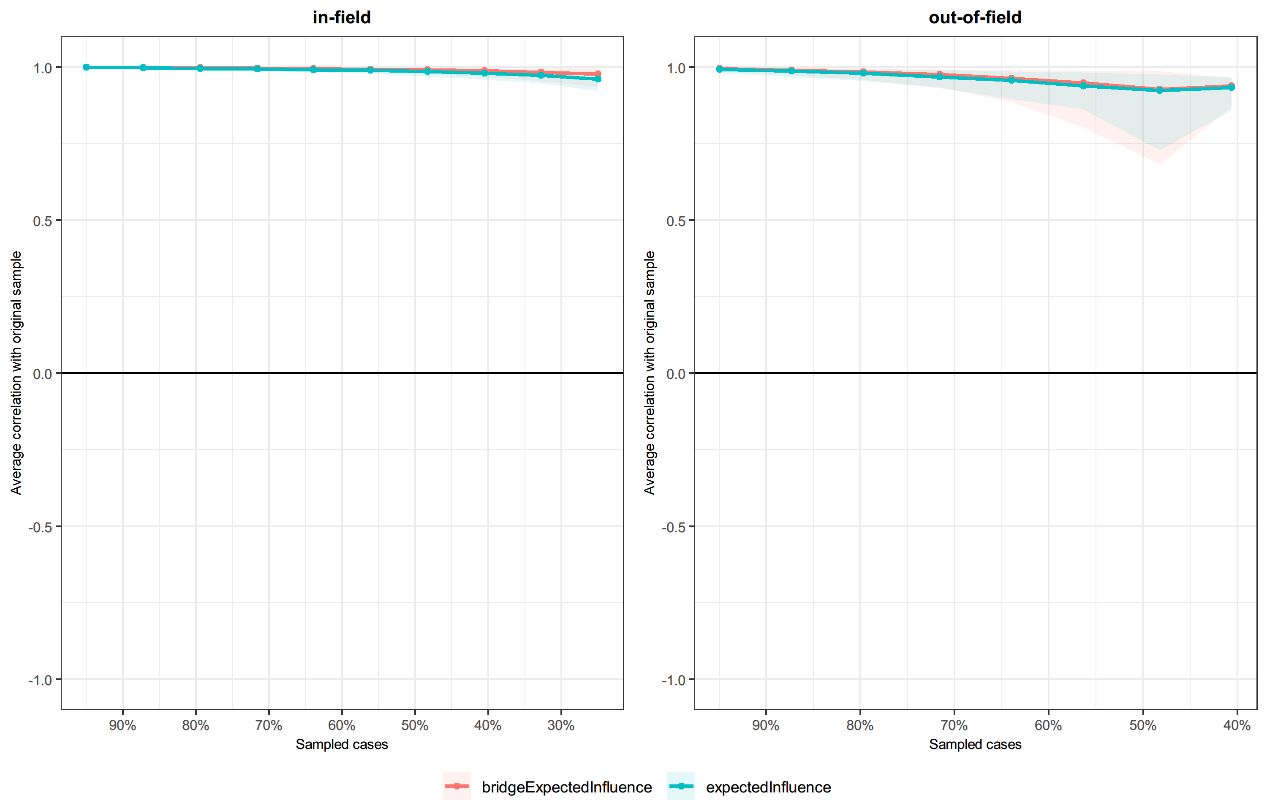


**Supplementary Figure 2.** Stability of EI and bridge EI for in-field and out-of-field math-major teachers.

## Supplementary Tables

**Supplementary Table 1.** Edge weights for in-field math-major teachers.

|  | JD1 | JD2 | JD3 | JR1 | JR2 | JR3 | JR4 | JR5 | JR6 | PR1 | PR2 | B1 | B2 | B3 |
| --- | --- | --- | --- | --- | --- | --- | --- | --- | --- | --- | --- | --- | --- | --- |
| JD1 |  | 0.00 | .04 | -.02 | 0.00 | .05 | .03 | -.03 | -.04 | 0.00 | 0.00 | .11 | 0.00 | 0.00 |
| JD2 |  |  | .40 | .04 | -.18 | .05 | .05 | -.01 | 0.00 | -.06 | .03 | .07 | .03 | .10 |
| JD3 |  |  |  | .08 | .06 | .03 | -.08 | 0.00 | 0.00 | .06 | .09 | .18 | 0.00 | 0.00 |
| JR1 |  |  |  |  | .24 | .07 | 0.00 | .10 | .09 | .03 | .13 | .05 | -.08 | 0.00 |
| JR2 |  |  |  |  |  | .13 | .19 | .21 | .09 | .11 | 0.00 | 0.00 | -.01 | 0.00 |
| JR3 |  |  |  |  |  |  | .28 | .16 | 0.00 | 0.00 | .03 | 0.00 | 0.00 | .02 |
| JR4 |  |  |  |  |  |  |  | .15 | .12 | 0.00 | 0.00 | -.08 | .01 | 0.00 |
| JR5 |  |  |  |  |  |  |  |  | .11 | .03 | 0.00 | -.02 | 0.00 | -.05 |
| JR6 |  |  |  |  |  |  |  |  |  | 0.00 | .02 | -.05 | -.20 | -.01 |
| PR1 |  |  |  |  |  |  |  |  |  |  | .66 | .07 | -.05 | 0.00 |
| PR2 |  |  |  |  |  |  |  |  |  |  |  | .12 | -.08 | 0.00 |
| B1 |  |  |  |  |  |  |  |  |  |  |  |  | .14 | .31 |
| B2 |  |  |  |  |  |  |  |  |  |  |  |  |  | .64 |
| B3 |  |  |  |  |  |  |  |  |  |  |  |  |  |  |

Note. JD1 = average teaching hours per week, JD2 = stress from student management, JD3 = stress from workload, JR1 = collaboration among teachers, JR2 = teacher-student relationship, JR3 = school resources, JR4 = school environment, JR5 = organizational justice, JR6 = job satisfaction, PR1 = classroom management efficacy, PR2 = instructional efficacy, B1 = emotional exhaustion, B2 = depersonalization, and B3 = diminished personal accomplishment.

**Supplementary Table 2.** Edge weights for in-field math-major teachers.

|  | JD1 | JD2 | JD3 | JR1 | JR2 | JR3 | JR4 | JR5 | JR6 | PR1 | PR2 | B1 | B2 | B3 |
| --- | --- | --- | --- | --- | --- | --- | --- | --- | --- | --- | --- | --- | --- | --- |
| JD1 |  | 0.00 | 0.00 | 0.00 | 0.00 | .01 | -.02 | -.06 | -.05 | 0.00 | .02 | .11 | 0.00 | 0.00 |
| JD2 |  |  | .22 | .02 | -.04 | .08 | 0.00 | 0.00 | -.13 | 0.00 | .04 | .07 | .04 | .06 |
| JD3 |  |  |  | 0.00 | 0.00 | -.06 | -.07 | 0.00 | 0.00 | .22 | .12 | .23 | 0.00 | 0.00 |
| JR1 |  |  |  |  | .22 | .12 | 0.00 | 0.00 | .03 | 0.00 | .14 | .05 | -.10 | 0.00 |
| JR2 |  |  |  |  |  | .06 | .14 | .33 | .04 | .07 | 0.00 | 0.00 | -.02 | -.01 |
| JR3 |  |  |  |  |  |  | .23 | .17 | 0.00 | 0.00 | .02 | 0.00 | .03 | .03 |
| JR4 |  |  |  |  |  |  |  | .12 | .04 | 0.00 | 0.00 | 0.00 | 0.00 | 0.00 |
| JR5 |  |  |  |  |  |  |  |  | .16 | .02 | .09 | 0.00 | -.04 | -.01 |
| JR6 |  |  |  |  |  |  |  |  |  | 0.00 | 0.00 | -.05 | -.14 | -.08 |
| PR1 |  |  |  |  |  |  |  |  |  |  | .51 | 0.00 | -.07 | -.03 |
| PR2 |  |  |  |  |  |  |  |  |  |  |  | .07 | 0.00 | 0.00 |
| B1 |  |  |  |  |  |  |  |  |  |  |  |  | .15 | .23 |
| B2 |  |  |  |  |  |  |  |  |  |  |  |  |  | .59 |
| B3 |  |  |  |  |  |  |  |  |  |  |  |  |  |  |

Note. JD1 = average teaching hours per week, JD2 = stress from student management, JD3 = stress from workload, JR1 = collaboration among teachers, JR2 = teacher-student relationship, JR3 = school resources, JR4 = school environment, JR5 = organizational justice, JR6 = job satisfaction, PR1 = classroom management efficacy, PR2 = instructional efficacy, B1 = emotional exhaustion, B2 = depersonalization, and B3 = diminished personal accomplishment.
